# Sensing R-Loop-Associated DNA Damage to Safeguard Genome Stability

**DOI:** 10.3389/fcell.2020.618157

**Published:** 2021-01-11

**Authors:** Carlo Rinaldi, Paolo Pizzul, Maria Pia Longhese, Diego Bonetti

**Affiliations:** Dipartimento di Biotecnologie e Bioscienze, Università di Milano-Bicocca, Milan, Italy

**Keywords:** R-loops, DNA damage, replication stress, ATM, ATR, DSBs

## Abstract

DNA transcription and replication are two essential physiological processes that can turn into a threat for genome integrity when they compete for the same DNA substrate. During transcription, the nascent RNA strongly binds the template DNA strand, leading to the formation of a peculiar RNA–DNA hybrid structure that displaces the non-template single-stranded DNA. This three-stranded nucleic acid transition is called R-loop. Although a programed formation of R-loops plays important physiological functions, these structures can turn into sources of DNA damage and genome instability when their homeostasis is altered. Indeed, both R-loop level and distribution in the genome are tightly controlled, and the list of factors involved in these regulatory mechanisms is continuously growing. Over the last years, our knowledge of R-loop homeostasis regulation (formation, stabilization, and resolution) has definitely increased. However, how R-loops affect genome stability and how the cellular response to their unscheduled formation is orchestrated are still not fully understood. In this review, we will report and discuss recent findings about these questions and we will focus on the role of ATM- and Rad3-related (ATR) and Ataxia–telangiectasia-mutated (ATM) kinases in the activation of an R-loop-dependent DNA damage response.

## Introduction

Genome integrity is constantly challenged by exogenous and endogenous events, the latter including essential cellular processes like DNA replication and transcription. As both DNA replication and transcription machineries might need to access the same DNA substrate during S phase, defects in their spatial and temporal coordination can lead to genome instability and ultimately contribute to the development of different diseases, including cancer.

During the last 20 years, several studies have pointed out that transcription plays both physiological and pathological roles not only through the production of mature RNA molecules but also through the generation of stable RNA–DNA hybrid intermediates. The term RNA–DNA hybrid refers to the base pairing of a single-stranded RNA molecule with a single DNA strand. Interestingly, this pairing is more stable than a DNA–DNA double strand (Roberts and Crothers, 1992; Sugimoto et al., 1995).

When formation of an RNA–DNA hybrid results in the displacement of the second DNA strand in the double helix, a three-stranded structure, called R-loop, is formed. While short RNA–DNA hybrids form transiently in each transcription bubble and during lagging-strand DNA synthesis, R-loops form *in cis* behind elongating RNA polymerases and their length spans from 0.1 to 2 kb (Ginno et al., 2012; Sanz et al., 2016; Malig et al., 2020). Importantly, some recent findings show that R-loops do not form *in trans* (Gómez-Gonzàlez and Aguilera, 2020).

R-loops are abundant; in fact, 5% of the human genome (and 8% of yeast genome) is occupied by these structures (Sanz et al., 2016; Wahba et al., 2016). Indeed, from yeasts to humans, R-loops generally accumulate at highly transcribed regions (e.g., rRNA and tRNA) and in specific genomic regions containing repetitive sequences (e.g., ribosomal DNA, centromeres, and telomeres). Furthermore, R-loops form at highly transcribed GC-rich sequences, and they have been associated with CpG island promoters as well as with terminator regions in mammals, where they contribute to regulate gene expression (Ginno et al., 2012, 2013; Sanz et al., 2016; Promonet et al., 2020). Even though R-loop formation is favored by an increasing GC-content of the template DNA strand (Ginno et al., 2012, 2013), this process is also influenced by both chromatin organization (García-Pichardo et al., 2017; Salas-Armenteros et al., 2017; Feldman and Peterson, 2019) and topology (Stolz et al., 2019). In particular, some findings suggest that DNA negative supercoiling is a key determinant for R-loop formation through DNA unwinding. Indeed, from bacteria to humans, the lack of DNA topoisomerase I (TOP1), which leads to increased DNA negative supercoiling, promotes R-loop accumulation (Massé and Drolet, 1999; Tuduri et al., 2009; El Hage et al., 2010; Manzo et al., 2018).

A programed formation of R-loops contributes to important cellular processes including transcription initiation and termination, mitochondrial DNA replication, immunoglobulin class switching, and epigenetic modifications. As several recent reviews describe the physiological roles of R-loops (García-Muse and Aguilera, 2019; Crossley et al., 2019; Brambati et al., 2020; Niehrs and Luke, 2020), we will not further review them.

R-loop levels and/or location are tightly regulated by different evolutionarily conserved pathways: (i) RNA processing factors involved in splicing, elongation, nuclear export, and degradation (Huertas and Aguilera, 2003; Li and Manley, 2005; Domínguez-Sánchez et al., 2011; Gómez-González et al., 2011); (ii) topoisomerases that relax DNA topology during transcription (Tuduri et al., 2009; El Hage et al., 2010); and (iii) chromatin remodelers that reduce RNA polymerase pausing (e.g., FACT complex) (Herrera-Moyano et al., 2014). In addition, RNase H enzymes (RNase H1 and H2 in eukaryotes) specifically degrade the RNA moiety of a RNA–DNA hybrid (Cerritelli and Crouch, 2009), and several factors that show RNA–DNA unwinding activities, like Sen1/SENATAXIN, Sgs1/BLM, Mph1/FANCM, and WRN, contribute to R-loop resolution genome-wide from yeasts to humans (Mischo et al., 2011; Skourti-Stathaki et al., 2011; Chang et al., 2017; García-Muse and Aguilera, 2019; Marabitti et al., 2019). Furthermore, defects in the homologous recombination proteins BRCA1 and BRCA2 (Bhatia et al., 2014, 2017; Hatchi et al., 2015), in the nucleotide excision repair (NER) proteins XPG and XPF (Sollier et al., 2014) and in the Fanconi anemia (FA) pathway (García-Rubio et al., 2015; Schwab et al., 2015; Bhatia et al., 2017), lead to R-loop accumulation, thus indicating that several DNA repair pathways contribute to R-loop regulation.

Besides their important physiological roles, R-loops are clearly emerging as potent sources of genome instability. Indeed, their altered homeostasis has been documented in several diseases, including neurological disorders and cancer (reviewed in Crossley et al., 2019; García-Muse and Aguilera, 2019; Brambati et al., 2020).

How can R-loops become detrimental for genome stability and contribute to the development of different pathologies? It is likely that harmful R-loops arise when their physiological turnover is impaired and/or when they abnormally form in particular genomic regions. As DNA transcription and replication share a common template, R-loops clearly represent an obstacle to DNA replication. Indeed, transcription–replication conflicts (TRCs) are considered to be the main source of R-loop-induced DNA damage and genome instability (Huertas and Aguilera, 2003; Prado and Aguilera, 2005; Gan et al., 2011; Helmrich et al., 2011). Moreover, R-loops have been shown to compromise genome stability by interfering with both transcription (Bonnet et al., 2017; Lang et al., 2017) and DNA damage repair processes (Ohle et al., 2016; Cohen et al., 2018; D’Alessandro et al., 2018; Lu et al., 2018).

In this review, we will focus on how R-loops threaten genome stability as well as on the interconnections between their regulatory mechanisms and the cellular response to either replication stress or DNA double-strand breaks (DSBs) formation. Moreover, we will report and discuss recent findings about the role of ATM- and Rad3-related (ATR) and Ataxia–telangiectasia-mutated (ATM) checkpoint kinases in protecting the genome by sensing aberrant R-loop formation.

## The Cellular Response to DNA Perturbations

Generation of DNA lesions and the presence of DNA replication stress both trigger the activation of sophisticated surveillance mechanisms, collectively called “DNA damage response” (DDR), which are essential to maintain genome stability and to inhibit pathological processes. Key players of the checkpoint responses are phosphatidylinositol 3-kinase-related protein kinases, including mammalian ATM (Ataxia–telangiectasia-mutated) and ATR (ATM- and Rad3-related), whose *Saccharomyces cerevisiae* orthologs are Tel1 and Mec1, respectively (reviewed in Blackford and Jackson, 2017).

Both ATM/Tel1 and ATR/Mec1 are activated by DNA damage, but their specificities are distinct. In fact, ATM/Tel1 is mainly activated by DSBs, whereas ATR/Mec1 responds to a broad spectrum of DNA perturbations that induce the generation of single-stranded DNA (ssDNA), including replication stress. Once activated, these kinases spread the signal to the downstream effector kinases CHK2 and CHK1 in mammals and Rad53 and Chk1 in *S. cerevisiae*. The main outcome of the DDR is the temporal coordination between DNA repair/replication resumption and cell cycle progression and, eventually, the induction of a permanent cell cycle arrest or of a programed cell death if the damage cannot be repaired.

### Replication Stress

Replication stress is a potent source of genome instability and a hallmark of cancer cells. Indeed, genome integrity is particularly at risk during S phase, especially when obstacles in the DNA template are present. For example, DNA secondary structures, DNA lesions, chromatin-bound protein complexes, and, interestingly, highly expressed genes are all causes of replication fork stalling (Zeman and Cimprich, 2014).

Replication stress triggers activation of a signaling cascade, known as the S-phase checkpoint. Stalled replication forks are characterized by stretches of ssDNA, which arise from the uncoupling of replicative polymerases and helicases and/or from nucleolytic processing of DNA. The ssDNA is bound with high affinity by the replication protein A (RPA) complex, which serves as a platform for the recruitment of numerous sensor proteins, including the heterotrimeric ring-shaped 9-1-1 complex (RAD9-RAD1-HUS1 in humans and Ddc1-Rad17-Mec3 in *S. cerevisiae*), which is loaded at the junctions between ssDNA and dsDNA by the RFC (replication factor C)-like clamp loader (RAD17-RFC2-5 in humans and Rad24-Rfc2-5 in *S. cerevisiae*) (Zou and Elledge, 2003; Flynn and Zou, 2011; Blackford and Jackson, 2017).

These events result in a full activation of ATR/Mec1, which spreads the checkpoint signal to CHK1 and CHK2/Rad53 kinases, thus leading to cell cycle arrest, stabilization of stalled replication forks, and inhibition of late origin firing. In doing so, the S-phase checkpoint promotes replication fork repair/restart and the completion of DNA replication from an adjacent origin (Segurado and Tercero, 2009; Flynn and Zou, 2011).

### DNA Double-Strand Breaks

One of the most cytotoxic forms of DNA damage is represented by the DSB. In fact, its defective repair can lead to a loss of genetic information and to chromosome rearrangements, which in turn can contribute to the pathogenesis of several human diseases, including cancer and neurodegenerative syndromes (Liu et al., 2012; O’Driscoll, 2012).

The repair of a DSB relies on either homology-dependent or homology-independent mechanisms. Homologous recombination (HR) is an error-free mechanism that requires a homologous template, usually a sister chromatid, to allow accurate repair of the DSB during the S and G2 phases of the cell cycle. Non-homologous end joining (NHEJ) is an error-prone mechanism that is active throughout the cell cycle and relies on the re-ligation of the two broken ends. While NHEJ requires no or limited processing of DNA ends, HR requires formation of 3’-ended single-stranded overhangs, through a process called DSB resection (Bonetti et al., 2018).

The highly conserved MRN/MRX complex (MRE11-RAD50-NBS1 in mammals and Mre11-Rad50-Xrs2 in *S. cerevisiae*) is rapidly recruited at DSBs, where it regulates DDR activation and promotes DSB repair. Furthermore, MRN/MRX is implicated in the recruitment and activation of the protein kinase ATM/Tel1 (Gobbini et al., 2016). Once activated by the presence of DSBs, ATM plays an intracellular signaling role, regulating cell cycle checkpoint activation and transcription and translation processes and modulating the local chromatin environment around DSBs to facilitate DSB signaling and repair (Blackford and Jackson, 2017; Bonetti et al., 2018; Casari et al., 2019).

## R-Loops as Sources of Genome Instability

**FIGURE 1:**
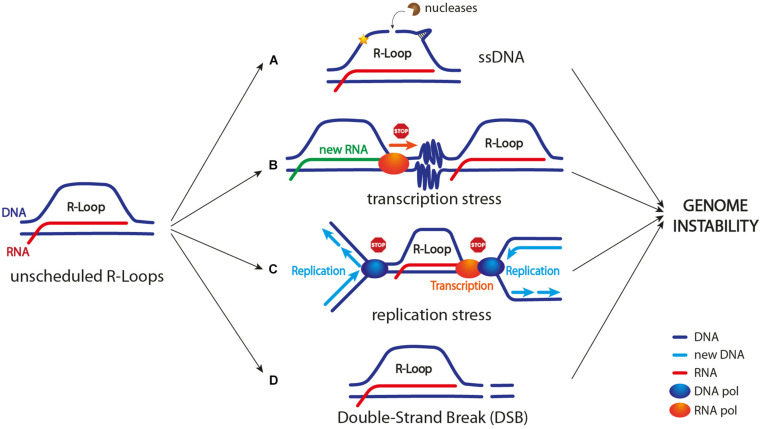
R-loop as a source of genome instability. Unscheduled R-loop formation can lead to genome instability in different ways. **(A)** Exposed ssDNA can be cleaved by different endonucleases leading to DNA breaks and/or mutagenic events (indicated by a yellow star); ssDNA can also adopt harmful secondary structures, including G-quadruplexes and hairpins. **(B)** R-loop accumulation, a stalled RNA polymerase in front of the transcription machinery, and/or R-loop-driven chromatin condensation (depicted as zig zag lines) can cause transcription block/slow down. **(C)** R-loop itself, a stalled RNA polymerase, chromatin condensation, and/or topological constrains can cause replication stress (see Figure 2 for more details). **(D)** R-loop might lead to DSB formation. Current models suggest that both replication forks collapse upon TRCs and R-loop processing by nucleases might lead to DSB formation (see Figure 3 for more details).

R-loop homeostasis is the result of a balance between their formation and removal throughout the genome. It is still unclear what exactly distinguishes a physiological from a pathological R-loop. Nonetheless, when their homeostasis is altered, at least in certain genomic regions (Costantino and Koshland, 2018), R-loops can turn into sources of DNA damage and genome instability by different ways, as described below and illustrated in Figure 1.

### R-Loop-Associated ssDNA

R-loop formation leads to the exposure of a ssDNA stretch on the non-template strand. Generally, ssDNA is vulnerable and it can turn into a source of both DNA mutagenesis and DNA breaks (Figure 1). For example, ssDNA in the R-loop can be targeted by DNA deaminases (e.g., AID in mammals) that convert cytidine to uracil. This event can lead to the formation of a DNA nick in case uracil is processed by the base excision repair machinery (BER). Furthermore, this DNA nick can be turned into a DNA DSB by the mismatch repair proteins, and this process is known to occur, for example, during immunoglobulin class switch recombination (CSR) (Muramatsu et al., 2000; Yu et al., 2003; Gómez-González and Aguilera, 2007). Moreover, R-loop-associated ssDNA can be cleaved by multiple endonucleases, including XPG, XPF, and FEN1, thus causing either DNA single-strand breaks (SSBs) or a DSB (Cristini et al., 2019; Marabitti et al., 2019; Figure 1). Lastly, ssDNA can adopt secondary structures, including G-quadruplexes and hairpins, that not only are prone to breakage but also represent obstacles to DNA replication (Freudenreich, 2018; Hegazy et al., 2020).

Importantly, it is still unclear how the R-loop-associated ssDNA is arranged *in vivo*. A study by Nguyen et al. (2017) suggests that it is coated by the RPA complex, which in turn acts as an R-loop sensor and promotes RNase H1 enzyme recruitment. The presence of RPA-coated ssDNA has been shown to trigger a specific R-loop-dependent ATR activation at centromeres during mitosis to promote faithful chromosome segregation (Kabeche et al., 2018). However, this ATR activation is non-canonical, because it occurs independently of DNA damage and replication stress, and there is no evidence for the recruitment of canonical ATR activators (e.g., the 9-1-1 complex).

### R-Loops as Sources of Transcription Stress

R-loops are well known for their physiological role as transcriptional regulators. Indeed, they are found at both promoters and terminators of several genes (Ginno et al., 2012; Chen et al., 2017; Hamperl et al., 2017; Promonet et al., 2020), where they regulate transcription initiation and ensure proper transcription termination, respectively. However, R-loops have also been shown to interfere with transcription (Bonnet et al., 2017; Lang et al., 2017), especially when their turnover is impaired.

Transcription stress arises when the RNA polymerase machinery either pauses, stalls, or backtracks due to obstacles or lesions in the DNA template. Interestingly, R-loops represent an obstacle to the transcription process too when their homeostasis is altered. However, it is still not clear whether transcription stress could be ascribed to the R-loop itself, to a stalled RNA polymerase, and/or to some chromatin modifications that are triggered by the R-loop (Figure 1).

DNA lesions can lead to transcription stress and activate a DNA damage response mainly involving the transcription-coupled nucleotide excision repair (TC-NER) pathway and in particular XPG and XPF nucleases (Gregersen and Svejstrup, 2018). However, it is still unknown whether an R-loop at stalled transcription sites could be resolved as a DNA lesion. Interestingly, a XPG- and/or XPF-dependent R-loop processing has been observed both in non-replicating and replicating cells, and this event has been associated with DSB formation and genome instability (Sollier et al., 2014; Cristini et al., 2019; Marabitti et al., 2019).

Finally, transcription stalling causes RNA polymerase backtracking, which in turn might be particularly dangerous, especially when a replication fork is approaching in the same direction as transcription. In fact, co-directional collisions between a replication fork and a backtracked RNA polymerase have been shown to cause chromosomal DSB formation (Dutta et al., 2011).

### R-Loops as Sources of Replication Stress

The transcription and replication machineries need to access the same template during S phase. Thus, they might collide in certain situations and/or at specific genomic regions. Notably, highly expressed human genes usually contain active replication origins in their promoter regions (Petryk et al., 2016; Chen et al., 2019), and long human genes require more than one cell cycle to be fully transcribed (Helmrich et al., 2011). Therefore, transcription–replication collisions during S phase are unavoidable (Figure 1). In addition, pausing, stalling, and backtracking of transcribing RNA polymerases further increase the chance of TRC and replication fork stalling (Hamperl and Cimprich, 2016). Stalled replication forks are particularly harmful because they are fragile structures that can either be processed by DNA nucleases or eventually collapse, thus resulting in chromosomal breakages and rearrangements (Zeman and Cimprich, 2014; Pasero and Vindigni, 2017). Importantly, TRCs are considered the main sources of R-loop-induced replication stress and DNA damage (Huertas and Aguilera, 2003; Prado and Aguilera, 2005; Gan et al., 2011; Helmrich et al., 2011; García-Muse and Aguilera, 2016).

Although we do not know exactly the frequency at which TRCs occur in normal cells, they likely become a problem in cells with an altered R-loop homeostasis. In fact, the lack of factors that regulate R-loop formation (e.g., RNase H enzymes and Sen1/SETX helicase) leads to replication stress and genome instability (Huertas and Aguilera, 2003; Gan et al., 2011; Aguilera and García-Muse, 2012; Costantino and Koshland, 2015, 2018). Interestingly, ectopic expression of RNase H enzymes relieves replication stress in cells accumulating R-loops, thus indicating that they physically interfere with the progression of replication forks (García-Muse and Aguilera, 2016; Kotsantis et al., 2016).

**FIGURE 2:**
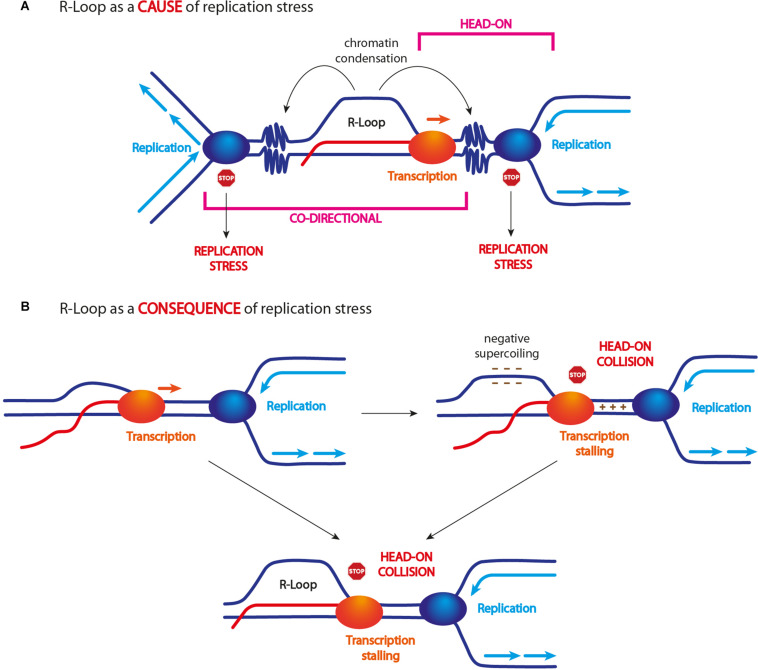
R-loop and replication stress. **(A)** Unscheduled R-loop formation causes replication fork arrest/slow down. This event has been ascribed to R-loop itself, a stalled transcription machinery, and/or R-loop-driven chromatin modifications, in particular its condensation. Some findings indicate that only head-on collisions are harmful for genome stability (see text for more details). **(B)** R-loop accumulation might also arise as a consequence of head-on TRCs. Head-on encounters can cause transcription arrest and R-loop accumulation. Head-on encounters can generate strong DNA positive supercoiling between the two approaching machineries and negative supercoiling behind them. Since negative supercoiling is known to promote R-loop formation/stabilization, this event might lead to their accumulation following head-on TRCs.

However, determining the exact cause of replication fork stalling is not straightforward. In fact, not only an R-loop *per se* but also a stalled RNA polymerase machinery may impede DNA replication and lead to further R-loop accumulation (García-Muse and Aguilera, 2016). Moreover, R-loops have been shown to trigger chromatin modifications, mainly including chromatin condensation (Castellano-Pozo et al., 2013; Al-Hadid and Yang, 2016; García-Pichardo et al., 2017; Figure 2), and this event seems to be a key requisite for compromising genome stability (García-Pichardo et al., 2017).

TRCs occur in two different modes: (i) when the replication and transcription machineries move in the same direction, it is defined as “co-directional collision” and (ii) when the two machineries move in opposite direction, it is defined as “head-on collision” (Figure 2). Although both types of TRCs can interfere with replication fork progression and stability, mainly head-on collisions have been shown to threaten genome stability (Prado and Aguilera, 2005; García-Muse and Aguilera, 2016; Hamperl et al., 2017; Promonet et al., 2020). Nonetheless, what exactly happens when DNA replication and transcription machineries collide in either orientation and how R-loops affect these events is not fully understood.

In addition, several findings suggest that R-loop levels are affected by TRC orientation. In particular, head-on collisions correlate with an increase of R-loop levels, while co-directional collisions do not (Hamperl et al., 2017; Lang et al., 2017; Figure 2). However, whether an R-loop represents the cause or the consequence of a head-on collision is still unclear. Studies in yeast suggest that an R-loop also forms in the context of co-directional collisions and it actually becomes a source of genome instability if stabilized (García-Rubio et al., 2018). Thus, R-loops seem to form independently of replication direction and, probably, they are not a consequence of TRCs. However, for still unclear reasons, they do not cause genome instability upon co-directional collisions.

It is likely that head-on moving machineries are more prone to collide, while co-directional collisions would occur only if the two machineries move at different speed. As the speed of replication and transcription machineries is comparable in eukaryotes, co-directional collisions are believed to be less frequent and to be promoted by additional events such as RNA polymerase stalling and/or backtracking. Moreover, it has been suggested that the replication machinery itself might resolve co-directionally formed R-loops during S phase through replicative helicases and/or replisome-associated factors (e.g., WRN, PIF1, and SETX) (Hamperl and Cimprich, 2016; Chang and Stirling, 2017).

By contrast, a head-on collision may lead to R-loop accumulation because the transcription process is blocked and the newly synthesized RNA cannot be released (Figure 2). Another model suggests that chromatin topology generated upon head-on TRCs might promote R-loop accumulation (Brambati et al., 2018; Chedin and Benham, 2020). Indeed, both transcription and replication machineries are known to accumulate DNA positive supercoiling in front of them, which might be exacerbated when the two machineries come in close proximity by opposite directions. The formation of DNA positive supercoils is known to generate an equal amount of negative supercoils in the opposite direction (Chedin and Benham, 2020), which are known to promote R-loop formation (Tuduri et al., 2009; El Hage et al., 2010; Figure 2).

In conclusion, R-loops clearly represent obstacles that can stall both transcription and replication processes, thus increasing the frequency and/or the negative effects of both co-directional and head-on collisions between the two machineries. Several lines of evidence indicate that, from bacteria to humans, genomes are organized to mainly have co-directionally moving transcription and replication machineries (Petryk et al., 2016; Merrikh, 2017; Chen et al., 2019; Promonet et al., 2020), thus suggesting that this general bias could help minimizing head-on collisions, R-loop accumulation, and genome instability.

### R-Loops and DNA DSBs

#### R-Loops as Sources of DSBs

**FIGURE 3:**
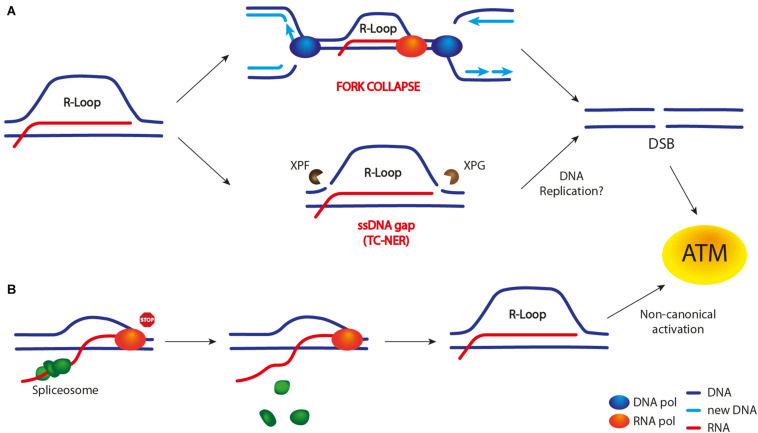
R-loop-dependent DSB formation and ATM/Tel1 activation. Unproper R-loop accumulation leads to both a DSB-dependent **(A)** and -independent **(B)** ATM activation. **(A)** Current models suggest that R-loop may lead to DSB formation either upon TRCs (in particular head-on collisions) and subsequent replication fork collapse or through R-loop cleavage by nucleases belonging to the TC-NER pathway (XPG and XPF in mammals). The latter event leads to SSB or ssDNA gap formation. However, whether the replication of this damaged template is required to convert it into a DSB is still unclear. **(B)** A DSB-independent ATM activation has been observed upon R-loop accumulation following transcription machinery stalling (e.g., by a DNA lesion) and spliceosome mobilization. The latter event is believed to promote R-loop formation/stabilization.

In replicating cells, R-loops are well known to impede the progression of replication forks (Gan et al., 2011). When stalled replication forks either are not stabilized or persist for extended periods of time, they might collapse, thus preventing replication restart and eventually leading to DSB formation (reviewed in Zeman and Cimprich, 2014). Moreover, in human cells, an altered R-loop homeostasis has been shown to cause DSB formation through the TC-NER pathway (Sollier et al., 2014; Figure 3). Importantly, recent findings support the idea that R-loops might promote DSB formation both by replication-dependent and -independent processes (Tresini et al., 2015, 2016; Cristini et al., 2019; Marabitti et al., 2019; Promonet et al., 2020). For example, in cells lacking the DNA topoisomerase 1 (TOP1), DSB frequency is increased at transcription termination sites (TTS) of highly expressed genes in an R-loop-dependent manner (Promonet et al., 2020). Interestingly, the same study shows that, at TTS, replication and transcription occur in opposite directions, thus suggesting that head-on collisions are the cause of DSB formation.

On the other hand, Cristini et al. (2019) have pointed out an R-loop-dependent, but replication-independent, process of DSB formation in non-replicating cells. As TOP1 is essential to relax supercoiled DNA during both transcription and replication, cells are constantly challenged by TOP1 cleavage complexes (TOP1cc) acting on DNA, which can eventually lead to transcription stalling and R-loop accumulation. Furthermore, removal of TOP1cc by the TDP1 excision pathway can generate a SSB. In the case that a second SSB is generated on the ssDNA of the R-loop structure (e.g., by XPG and XPF nucleases), a DSB is formed.

#### R-Loops and DSB Repair

Recent studies in yeast and mammals have implicated transcription and RNA–DNA hybrid formation in DSB signaling and repair. Different research groups have shown that pre-existing transcripts and, interestingly, *de novo*-synthesized non-coding RNAs promote both the efficient signaling and repair of the DSB (Francia et al., 2012, 2016). Moreover, a transient formation of RNA–DNA hybrids at DSB sites seems to be a key step in DSB repair (Ohle et al., 2016; D’Alessandro et al., 2018). Thus, DSB repair is another important process through which R-loops/RNA–DNA hybrids can impact on genome stability.

One important class of RNA molecules involved in DSB response are DNA-damage response RNAs (DDRNAs), which show the same sequence as damaged DNA and are generated after processing by the RNA interference machinery factors DICER and DROSHA. It has been shown that DDRNAs are required for a full activation of the DDR response (Francia et al., 2012; d’Adda di Fagagna, 2014). Moreover, similar very short ncRNA species, named diRNAs, contribute to DSB repair by HR (d’Adda di Fagagna, 2014; Gao et al., 2014). While DDRNAs map very close to DNA ends (Francia et al., 2012), diRNAs are generated starting from a few hundred nucleotides away from the DSB end (Wei et al., 2012). Thus, sequence-specific RNAs may act as guides for the localization and/or activation of several factors, including DDR and DNA repair proteins.

In addition, findings in *Schizosaccharomyces pombe* and mammalian cells (Ohle et al., 2016; D’Alessandro et al., 2018; Cohen et al., 2018; Lu et al., 2018) indicate that RNA–DNA hybrids are formed at DSBs and that they play an important role in promoting DSB repair by HR. Indeed, these RNA–DNA hybrids contribute to the recruitment of the HR proteins BRCA1, BRCA2, and RAD51. Despite this positive role in promoting accurate DNA repair, persistence of these RNA–DNA hybrids seems to exert negative effects by interfering with proper loading of HR factors, like RPA (Ohle et al., 2016) and RAD51 (Cohen et al., 2018). Interestingly, BRCA2 directly interacts with RNase H2, mediates its localization to the DSB in the S/G2 cell-cycle phase, and controls RNA–DNA hybrid resolution (D’Alessandro et al., 2018). Moreover, in both yeasts and humans, senataxin/SEN1 is recruited to DSBs, where it regulates the repair process (Cohen et al., 2018; Rawal et al., 2020). Thus, formation of RNA–DNA hybrids at DSBs is tightly controlled and has to be a transient event.

## The Cellular Response to Altered R-Loop Homeostasis

### R-Loop-Dependent ATM/Tel1 Activation

ATM/Tel1 is one of the apical kinases orchestrating the DDR at DSBs. Therefore, it is not surprising that several lines of evidence indicate R-loop-dependent ATM activation mechanisms. However, several questions are still open: (i) is ATM activated by an R-loop-induced DSB or by other signals? (ii) Is DNA replication required to activate ATM? (iii) How can transcription and R-loops be both a cause and a consequence of DSB formation?

The first evidence of an R-loop-dependent ATM activation comes from a study showing that co-directional TRCs specifically activate ATM, while head-on TRCs specifically activate ATR (Hamperl et al., 2017). However, the nature of this bias is still unknown. A possible explanation could be that, as previously mentioned, chromosomal DSBs arise as a consequence of co-directional conflicts occurring upon collisions with a backtracked or stalled transcription machinery (Dutta et al., 2011). Nonetheless, whether head-on collisions might lead to ATM activation is still unclear.

Interestingly, an R-loop-dependent ATM activation was observed in replicating cells lacking the WRN helicase, and this event is crucial to limit genome instability (Marabitti et al., 2019). Importantly, ATM activation is triggered by R-loop accumulation and it requires R-loop processing by the TC-NER pathway, i.e., the XPG nuclease (Figure 3). In fact, the effects caused by ATM deficiency can be rescued both by reducing R-loop levels and by depleting XPG nuclease.

By contrast, a study from Tresini et al. (2015) shows that ATM can be activated independently of DNA replication and DSB formation. In fact, ATM activation occurs in non-replicating cells. Moreover, it occurs when specific transcription-blocking DNA lesions lead to spliceosome mobilization, followed by R-loop accumulation/persistence (Tresini et al., 2015). Active ATM promotes further spliceosome displacement and the activation of the DDR response (Figure 3), which also influences gene expression and alternative splicing genome-wide. The same authors also show that ATM activation is DSB-independent and it occurs in a non-canonical manner, without the need of the MRN complex (Tresini et al., 2016).

As previously mentioned, a study from Cristini et al. (2019) demonstrates an R-loop-dependent but replication-independent DSB formation mechanism in non-replicating cells. However, whether these DSBs activate ATM has not been reported.

In conclusion, it is not clear how exactly R-loops activate the ATM kinase. Intriguingly, TRCs and DSBs are not sources of ATM activation in all reported studies. It is important to mention that, although ATM is primarily activated by a DSB, the specific signals that activate this kinase are still not fully understood. For example, ATM activation upon oxidative stress does not depend on either DSBs or the MRN complex (Guo et al., 2010). It is tempting to speculate that, in both replicating and non-replicating cells, R-loop persistence, either because of transcription stalling or defects in factors involved in their regulation (e.g., WRN), might lead to DSB formation through an R-loop processing by nucleases rather than upon replication forks collapse. By contrast, R-loop cleavage and replication fork collapse might lead to ATM activation through two distinct mechanisms (Figure 3).

It is worth mentioning a recent study showing that lack of ATM/Tel1 only causes a slight increase in R-loop levels genome-wide, compared to the lack of ATR (Barroso et al., 2019). Moreover, the lack of ATM causes neither significant defects in DNA replication progression nor an increase in R-loop-dependent DSB formation. However, DSBs accumulate genome-wide in cells lacking ATM. Barroso and colleagues suggest that the mild accumulation of R-loops in cells lacking ATM might be a consequence of unrepaired DSBs rather than the source of DSBs. Thus, whether ATM might promote R-loop resolution is still unclear. The same study also shows that ATM depletion leads to chromatin condensation, i.e., histone H3-S10 phosphorylation and, to a less extent, H3-K9 methylation. Interestingly, H3-S10 phosphorylation was previously shown to be strongly associated with R-loop-driven genome instability (García-Pichardo et al., 2017), thus making the uncovering of the links between ATM, R-loops, and chromatin state intriguing.

### R-Loop-Dependent ATR/Mec1 Activation

The interconnections between ATR/Mec1 and transcription have been suggested by different studies. In yeast, Mec1 and the chromatin remodeling complex INO80 were shown to inhibit transcription proximal to early firing origins in the presence of replication stress, thus limiting TRCs (Poli et al., 2016). Moreover, Mec1/ATR was shown to promote the release of actively transcribed genes from nuclear envelope, thus releasing topological constrains and protecting fork stability (Bermejo et al., 2011). Interestingly, the ATR pathway is involved in maintaining the stability of common fragile sites (CFS), which are specific genomic regions that are difficult to replicate and prone to breakage upon replication stress (Casper et al., 2002; Barlow et al., 2013). Since some CFSs correspond to long or highly transcribed genes that tend to accumulate R-loops (Helmrich et al., 2011; Groh et al., 2014), these data suggest a possible role for ATR in both sensing and regulating R-loops, at least in certain genomic regions including CFS.

**FIGURE 4:**
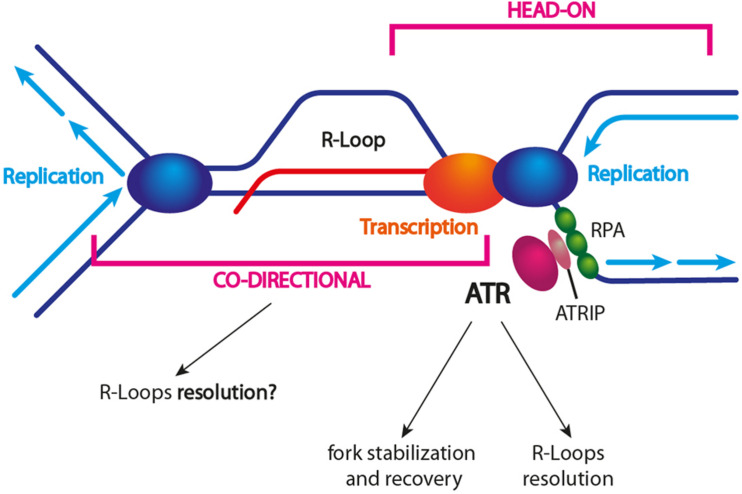
R-loop-dependent ATR/Mec1 activation. TRCs caused by unscheduled R-loop formation trigger ATR and activation of the S-phase checkpoint. The current model suggests that head-on collisions trigger ATR and DDR activation, which in turn promote fork protection and restart. It is still unclear whether co-directional collisions might activate ATR. The exact mechanism of R-loop resolution is still unclear as well. Different factors (e.g., helicases) might be actively recruited to a stalled fork other than being associated with an incoming (co-directional) fork.

ATR/Mec1 activation is observed upon replication stress. As R-loops represent obstacles to replication, ATR/Mec1 activation might be triggered by R-loop-driven stalled replication forks (Figure 4). Indeed, in both yeast and humans, an ATR/Mec1 response has been detected during S phase in cells harboring high R-loop levels (Gómez-González et al., 2009; Matos et al., 2020). Moreover, ATR was shown to be activated specifically in the presence of head-on TRCs (Hamperl et al., 2017). Interestingly, when cells are depleted of either ATR, CHK1, or components of the 9-1-1 complex, R-loops accumulate and replication slows down genome-wide. These observations confirm that R-loops are sources of replication stress and that the ATR pathway is required to suppress their accumulation (Barroso et al., 2019).

How might ATR promote genome stability in the presence of R-loop-associated replication stress? First, it might trigger the recruitment of specific R-loop resolving factors to a stalled replication fork (Figure 4). For example, the DDX19 nucleopore-associated RNA helicase has been shown to reduce R-loops and to relieve TRCs by an ATR-dependent mechanism (Hodroj et al., 2017). Moreover, SETX recruitment to sites of RNA–DNA hybrid-associated replication stress requires the DDR response, even though the ATM and DNA-PK kinases are also important (Yüce and West, 2013). Recent findings by Matos et al. (2020) confirm that an altered R-loop homeostasis activates the ATR-CHK1 pathway in a replication-dependent manner. In contrast to ATR activation by the replication inhibitor hydroxyurea (HU), R-loop-induced ATR activation requires the MUS81 endonuclease. Once activated, ATR protects the genome against R-loop-associated DNA damage through several mechanisms: (i) it reduces TRC frequency by still unknown mechanisms, (ii) it promotes replication fork recovery, and (iii) it enforces a G2/M checkpoint arrest. In addition, ATR prevents the excessive cleavage of reversed replication forks by MUS81, thus revealing an ATR-mediated feedback loop that fine-tunes MUS81 activity at R-loop-impeded replication forks (Matos et al., 2020).

It is known that, when a replication fork becomes dysfunctional, the completion of DNA replication could be ensured by a converging functional fork or, alternatively, by a fork restart that requires the homologous recombination pathway (Zeman and Cimprich, 2014; Pasero and Vindigni, 2017). Interestingly, recent data show that MUS81 and homologous recombination promote replication completion in response to replication stress by providing fork protection until a functional fork comes, rather than promoting the restart of DNA synthesis from the stalled fork itself (Pardo et al., 2020). Thus, ATR and MUS81 might be involved in this mechanism in response to R-loop-mediated replication stress.

It has been shown that ATR activation occurs in the presence of head-on TRCs (Hamperl et al., 2017; Promonet et al., 2020). Since it has been suggested that co-directional TRCs lead to R-loop removal, it is tempting to speculate that an incoming co-directional replication fork might help to resolve the stress, i.e., the R-loop, and to complete replication at head-on collision sites that are stabilized by ATR/Mec1 (Figure 4).

## R-Loop and Diseases

R-loops are clearly emerging to have a central role in cell biology, not only for their physiological roles but also for their pathological implications. Several studies point out that R-loops generate genome instability by affecting different cellular processes, such as DNA transcription, replication, and repair. Moreover, an altered R-loop homeostasis has been documented in several diseases, including neurological disorders and cancer (reviewed in Crossley et al., 2019; García-Muse and Aguilera, 2019; Brambati et al., 2020). It is not surprising then that the list of factors associated with human diseases and involved in R-loop regulation is continuously growing.

Just to mention some of them, BRCA1 and BRCA2 proteins, which are associated with breast and ovarian cancer development, have a role in R-loop regulation at promoters and terminators of transcribed genes and at DSBs (Bhatia et al., 2014; Hatchi et al., 2015; D’Alessandro et al., 2018; Shivji et al., 2018). Interestingly, mutations in BRCA1 cause R-loop accumulation at specific genes and an altered transcription rate, and these events seem to be directly implicated in tumorigenesis (Zhang et al., 2017; Chiang et al., 2019). In BRCA2-deficient cells, RNase H1 overexpression reduces formaldehyde-induced replication fork stalling as well as structural chromosomal aberrations formed under these conditions, thus suggesting that R-loops contribute, at least partially, to the pathogenic effects of BRCA2 inactivation (Tan et al., 2017).

Another example of the interconnections between R-loops and cancer is related to the AID-mediated mutagenesis during immunoglobulin class switching. In fact, this process has been implicated in chromosomal translocations between the Ig loci and other active genes, leading to oncogenic gene expression (Robbiani and Nussenzweig, 2013). Interestingly, R-loops have also been mapped at common translocation partners of Ig genes, in particular the oncogene *c-MYC* (Yang et al., 2014).

The R-loop resolving SETX helicase is associated with neurological disorders like AOA2 (ataxia with oculomotor apraxia type 2) and ALS4 (amyotrophic lateral sclerosis 4). AOA2 is associated with loss-of-function mutations in the SETX gene (Moreira et al., 2004), and cells from AOA2 patients or depleted for SETX show R-loop accumulation, altered gene expression, and increased DNA damage and cell death (Suraweera et al., 2007, 2009; Becherel et al., 2015). On the other hand, studies performed with cells from patients suffering ALS4 identified missense mutations in SETX gene (Chen et al., 2004). These mutations are gain-of-function mutations and correlates with decreased R-loop levels and altered chromatin methylation over more than 1,000 genes, which in turn likely leads to a change in their expression (Grunseich et al., 2018). It is worth pointing out that the SETX helicase is also recruited by BRCA1 to limit R-loops and DNA damage at gene terminators (Hatchi et al., 2015), thus indicating interconnections between these pathways.

Finally, mutations inactivating the RNase H2 enzyme are associated with the rare Aicardi-Goutières inflammatory syndrome (AGS) and with systemic lupus erythematosus (Crow et al., 2006; Gunther et al., 2015), both disorders being characterized by an abnormal innate immune response. Interestingly, AGS fibroblasts display pronounced RNA–DNA hybrid accumulation and global loss of DNA methylation genome-wide (Lim et al., 2015). However, despite of the progress in the field, the exact contribution of an altered R-loop homeostasis to the development of several diseases is unfortunately still unclear.

## Discussion

In the last years, our knowledge about R-loops and about the outcomes of their altered homeostasis on genome stability has grown exponentially. Important progresses have been made in the identification of proteins regulating R-loop formation, stabilization, and resolution and new players are continuously identified. However, several critical questions remain to be addressed. For example, one important gap to be filled in concerns R-loop sensing. In particular, it is important to better understand how the cellular response to unscheduled/aberrant R-loops is orchestrated and which pathways are activated in order to protect genome stability. Moreover, more insights are necessary into the molecular mechanisms triggering R-loop-mediated genome instability and into the interconnections between R-loops, TRCs (both head-on and co-directional), and replication stress. In particular, since some findings suggest that R-loops generate genome instability independently of DNA replication, this aspect may be connected to neurological disorders, as these pathologies affect non-dividing neuronal cells.

Another important aim is to untangle the controversial roles of R-loops in DNA DSB formation and repair. In fact, conflicting results have been obtained regarding formation and function of RNA–DNA hybrid intermediates at DSBs. In particular, there is still an ongoing debate on whether the pre-existing transcriptional state of a damaged locus could be a key determinant of R-loop formation and which RNA species actually form a hybrid with DNA. i) Is R-loop formation a feature of all DSBs or only of those occurring in actively transcribed loci? ii) Which is the source of RNAs: pre-existing transcription, *de novo* transcription, or both? iii) Are R-loops promoting or inhibiting DNA repair? This scenario is further complicated by the evidence that pre-existing transcription (and likely R-loop formation) is inhibited by the DDR when a DSB occurs in actively transcribed regions in mammals (Shanbhag et al., 2010; Harding et al., 2015). These data appear to be in contrast with a co-existing *de novo* transcription at the DSB and this paradox needs to be clarified. Interestingly, Bader and Bushell (2020) propose that pre-existing transcription at DSBs is shut down and R-loops are removed if present, but pre-existing RNA species, rather than *de novo* ones, are important for the formation of new RNA–DNA hybrids in close proximity of the break. Thus, the molecular mechanisms of R-loop regulation at DSBs are extremely complex, and we have so far managed to discover only the tip of the iceberg.

Finally, another important and only partially answered question concerns how exactly an R-loop becomes unscheduled, aberrant, or pathological? To address this question, it will be fundamental to determine the location, the frequency, and the half-life of R-loops genome-wide and to compare different conditions and cell types in a quantitative way. Very recently, important improvements have been made in techniques for R-loop detection. For example, “footprinting” methods represent powerful tools to determine the exact position and length of R-loops in the genome (Malig et al., 2020). In addition, techniques determining R-loop frequencies and their half-lives have been definitely improved (Crossley et al., 2020; Malig et al., 2020). Importantly, significative upgrades have also been made in allowing precise quantitative comparisons of R-loop levels genome-wide under different conditions, especially in pathological vs. healthy conditions (Crossley et al., 2020). Altogether, these methods will help us to determine the pathogenic landscape of R-loops.

## Author Contributions

DB and ML conceived the idea. CR and DB wrote the manuscript. DB, CR, and ML revised and edited the manuscript. CR and PP created the figures. All authors contributed to the article and approved the submitted version.

## Conflict of Interest

The authors declare that the research was conducted in the absence of any commercial or financial relationships that could be construed as a potential conflict of interest.
